# Genetic mating system and mate selection in smallmouth bass

**DOI:** 10.1002/ece3.3423

**Published:** 2017-09-20

**Authors:** Ryan P. Franckowiak, Mark S. Ridgway, Chris C. Wilson

**Affiliations:** ^1^ Environmental and Life Sciences Graduate Program Trent University Peterborough ON Canada; ^2^ Harkness Laboratory of Fisheries Research Ontario Ministry of Natural Resources and Forestry Aquatic Research and Monitoring Section Trent University Peterborough ON Canada; ^3^ Conservation and Genetics Laboratory Ontario Ministry of Natural Resources and Forestry Aquatic Research and Monitoring Section Trent University Peterborough ON Canada

**Keywords:** maternal reconstruction, molecular pedigree, monogamy, multiple mating, sexual selection, sibship reconstruction

## Abstract

Mating systems are an important factor influencing the variance in reproductive success among individuals within natural populations and thus have important ecological and evolutionary implications. We used molecular pedigree reconstruction techniques with microsatellite DNA data to characterize the genetic mating system and mate selection in adult smallmouth bass spawning in Lake Opeongo. The genetic mating system of smallmouth bass in this system can be characterized as predominantly monogamous with a low rate of polygynandry particularly among larger individuals. Iteroparous individuals showed a complete absence of interannual mate fidelity, presumably due to the low annual return rate of spawning adults. Within a season, individuals from both sexes pursued additional mating opportunities with males showing greater variance in mate number than females. Female mate selection appeared to be largely random with little evidence for elevated levels of inbreeding in this population. Multiple mating females pursued additional males to whom they were less related than the first male with which they spawned within a given season, however, this pattern varied among years. The mating pattern observed in this population would likely limit the strength of sexual selection and thus could account for the lack of sexual dimorphism and the absence of alternative reproductive tactics in this species.

## 1 INTRODUCTION

Animal mating systems are exceptionally diverse, and considerable effort has been undertaken to document and describe variation in mating systems among taxonomic groups, to identify the factors leading to and maintaining this variation, and to understand the ecological and evolutionary implications of different mating systems (Davies, 1991; Emlen & Oring, 1977; Shuster & Wade, 2003). Due to the considerable complexity of animal mating systems and the degree to which they can vary both within and among populations, it can be exceedingly difficult to accurately identify mating behavior in the absence of genetic data (Klug, 2011). The development of highly variable genetic markers has contributed greatly to the study of mating systems and social structure in a wide range of species, often revealing a higher degree of promiscuity than was apparent from behavioral observations alone (DeWoody & Avise, 2001; Gibbs & Weatherhead, 2001; Griffith, Owens, & Thuman, 2002; Ross, 2001).

Considerable lability in mating pattern is expected between populations exposed to different environmental or demographic conditions (Shuster & Wade, 2003). The most influential factors include the availability and distribution of critical resources and the spatial and temporal distribution of sexually receptive mates (Emlen & Oring, 1977; Shuster & Wade, 2003). Changes in the spatial or temporal distribution of a critical resource from 1 year to another or from one area to another are expected to alter the environmental potential for polygamy (Emlen & Oring, 1977; Shuster & Wade, 2003). With increasing variance in habitat quality, there exists a greater potential for individuals to monopolize resources, and thus a greater environmental potential for polygamy, and sexual selection is expected to intensify (Emlen & Oring, 1977; Shuster & Wade, 2003). However, changes in the energetic cost of resource or mate monopolization resulting from changes in population density and the length of breeding season are expected to influence the ability of individuals to take advantage of the environmental potential for polygamy.

Fish species exhibit the full range of mating systems, from strict monogamy to polygyny, polyandry, and polygynandry (Avise et al., 2002), and thus provide a rich conceptual arena for examining the causal factors regulating genetic mating systems and reproductive tactics (Avise et al., 2002). Parental care is widespread in fish and it represents an important component of their mating systems (Wootton & Smith 2014), but which sex provides care often differs widely among families (DeWoody & Avise, 2001; Reynolds, Goodwin, & Freckleton, 2002). Care decisions are influenced by the availability of mates and the level of care required for offspring production, and thereby provides a natural link between the theories of sexual conflict and parental investment (Kokko & Jennions, 2008). In the bony fishes, approximately 21% of taxonomic families include species in which adults provide parental care, and in nearly 70% of those families, the male is the primary or exclusive care provider (Avise et al., 2002; Blumer, 1979, 1982). Paternal care increases a male's certainty of paternity (Ah‐King, Kvarnemo, & Tullberg, 2005; Kvarnemo, 2006) and some females prefer to spawn with males that are already providing care (Hale & St Mary, 2007; Pruett‐Jones, 1992; Reynolds & Jones, 1999).

The smallmouth bass (*Micropterus dolomieu*) are a territorial, nest building, freshwater fish species native to lakes and streams throughout central and eastern North America (Scott & Crossman, 1973). During spring, mature males move into the littoral zone when water temperature is approximately 15°C, excavate a saucer‐shaped nest in the substrate, engage in extensive courtship behavior away from and near the nest, spawn via external fertilization, and remain at the nest as the sole provider of parental care until the offspring disperse (Ridgway, 1988; Wiegmann, Baylis, & Hoff, 1992). Smallmouth bass are generally considered to be monogamous (Wiegmann et al., 1992), solitary nesters (Vogele, 1981), that do not exhibit alternative reproductive strategies (i.e., sneaker or satellite males) often displayed by other centrarchid species (Ridgway, Goff, & Keenleyside, 1989). Spawning adults are thought to exhibit size assortative mating (Wiegmann et al., 1992), with larger individuals spawning earlier in the season than smaller individuals (Ridgway, Shuter, & Post, 1991). Males display strong nest site fidelity (Ridgway, MacLean, & McLeod, 1991; Ridgway, Shuter, Middel, & Gross, 2002) and are presumed to exhibit strong philopatry to natal nesting areas (Gross, Kapuscinski, & Faras, 1994). These behaviors are expected to concentrate relatives during reproduction and thereby increase the probability of mating between closely related individuals.

The purpose of this study was to examine the causal factors regulating genetic mating systems and reproductive tactics in a centrarchid species. To this end, we characterized the genetic mating system of smallmouth bass in Lake Opeongo. To overcome the logistical challenges of identifying and capturing nesting pairs of adults in the field, we applied molecular pedigree reconstruction approaches with microsatellite genotype data to determine sibship relationships between swim‐up fry within and among sampled nests, confirm the paternity of nest guarding males, and reconstruct the genotype (and thus the identity) of spawning females, which were not directly sampled. Male and female genotypes were then used to track individuals during multiple successive breeding seasons to determine the extent of interannual mate fidelity and also the prevalence of multiple mating by both males and females within each respective season. Lastly, we examined the genetic relatedness of male–female spawning pairs to assess whether female smallmouth bass actively avoid mating with close relatives.

## 2 MATERIALS AND METHODS

### 2.1 Study system

Lake Opeongo (45″42′N, 78′22′W) is a multibasin, oligotrophic lake located in the highlands of Algonquin Provincial Park, Ontario, Canada. The lake has a surface area of 58.6 km^2^, a maximum depth of 51.8 m, a mean depth of 14.8 m, and a mean Secchi depth of 6 m (Martin & Fry, 1972, 1973). The lake consists of three limnologically distinct basins (South Arm, North Arm & East Arm) and two smaller bays (Sproule Bay & Annie Bay) each separated by a shallow sill and/or a restricted channel (Finlay, Cyr, & Shuter, 2001). The shoreline consists mostly of rock and gravel with some areas of sand and is largely undeveloped with the exception of a single access point located in the southeastern end of Sproule Bay. Smallmouth bass are not native to the lake but instead were established as a result of historical stocking that occurred throughout the region, with the first recorded observation in Lake Opeongo reported in 1928 (Christie, 1957; Martin & Fry, 1972; Shuter & Ridgway, 2002). Since then, the population continued to grow and expand, becoming fully established by the 1960s (Martin & Fry, 1972). This population has been studied extensively and these studies have provided valuable insights into numerous aspects of smallmouth bass reproductive ecology and behavior (see Ridgway et al., 2002; and citations therein).

### 2.2 Field surveys

**Figure 1:**
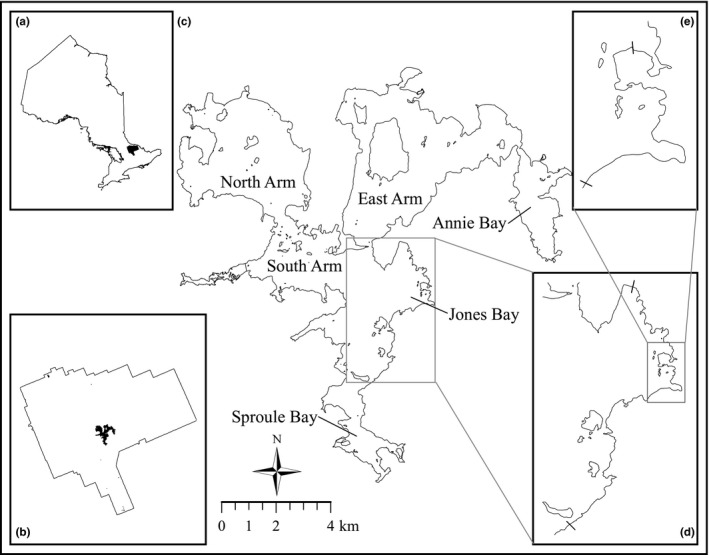
Map of study area including (a) Ontario provincial boundary showing the location of Algonquin Provincial Park, (b) Algonquin Provincial Park boundary showing the location of Lake Opeongo, (c) Lake Opeongo boundary, (d) South Arm shoreline (14.8 km) surveyed in 2012 and 2013, and (e) Jones Bay shoreline (5.7 km) surveyed in 2011 and 2014. Hash mark along the shoreline of the South Arm and Jones Bay define the extent of the area sampled (including islands)

We conducted snorkeling surveys from late May to early July, 2011–2014, along the windward (i.e., eastern) shoreline of the South Arm, where the majority of spawning activity occurs within the basin (Rejwan et al. 1997). A 14.6 km section of shoreline (including Jones Bay) was surveyed during the 2012 and 2013 spawning seasons, whereas in 2011 and 2014, the study area was restricted to a 5.8 km section of shoreline within Jones Bay (Figure 1). We marked each nest location with a uniquely numbered polyvinyl chloride (PVC) cylinder placed near the nest perimeter, and the GPS position of each nest was recorded. Snorkel surveys were repeated every 3–5 days to track the progression of previously marked nests and to identify any new nests or nests overlooked in previous surveys. The seasonal phenology of nesting males (Ridgway, Shuter, et al. 1991) allowed us to distinguish between the first and any subsequent mates selected by females who participated in multiple mating each year (see Sections 3 and 4).

We captured nest guarding males using recreational fishing equipment either from a boat or by swimmers in the water as previously described by Ridgway, Shuter, et al. 1991; Ridgway, MacLean, et al. 1991. Each male was tagged with uniquely numbered T‐bar anchor tags (Hallprint, Victor Harbour, South Australia), which were placed beneath the soft dorsal fin for individual identification in the field. We measured the fork length of each male, collected scales and a dorsal spine for aging purposes, and took a small caudal fin clip for genetic analysis before returning each individual to its nest. We used small aquarium dip nets to collect swim‐up fry (~ 8–12 per nest) from each surviving brood (with the exception of the 2011 spawning season) just prior to them dispersing from the nest. Samples from each brood were stored separately in individually labeled scintillation vials (Fisher Scientific Company, Ottawa, Canada) and preserved with 95% ethanol.

### 2.3 Genetic analysis

We extracted DNA from fin tissue collected from each adult male and whole swim‐up fry using a lysis buffer extraction protocol described by Wilson, Lavender, and Black (2007), with the resulting DNA pellet being resuspended in 200 μl of low EDTA TE buffer (10 mM Tris, 0.1 mM EDTA, pH 7.5–8.0). The quality of the extracted DNA was determined by size fractionation on an agarose gel and quantified using the Thermo Scientific^™^ GeneRuler^™^ 100 bp DNA Ladder (Fisher Scientific Company, Ottawa, Ontario, Canada) run on the same gel. Due to the general high yield, we further diluted the extracted DNA by adding 1 μl eluted DNA to 30 μl low EDTA TE buffer prior to use. Each individual was genotyped at 20 microsatellite loci amplified via the polymerase chain reaction (PCR) in five separate multiplex reactions (Table S1). PCR amplifications were performed on a Mastercycler^®^ Pro thermocycler (Eppendorf Canada, Mississauga, ON). The resulting microsatellite amplicons were run on an ABI 3730xl DNA Analyzer (Applied Biosystems, Inc., Burlington, ON) with GeneScan^™^ 500LIZ^™^ (Applied Biosystems) as the internal size standard and scored using GeneMapper v4.0 (Applied Biosystems). Fragment sizing was confirmed by manual proofreading.

### 2.4 Pedigree reconstruction and mating system

We used the full‐pedigree likelihood method of Wang and Santure (2009) implemented in COLONY v2.0 (Jones & Wang, 2010) to simultaneously infer sibship of swim‐up fry, confirm (i.e., assign) paternity of nest guarding males, and reconstruct maternal genotypes. This approach uses a simulated annealing technique to iteratively search multilocus genotype data for the best pedigree configuration with maximum likelihood and estimates confidence using a Bayesian averaging procedure based on a 1,000 plausible pedigree configurations archived during the simulated annealing procedure (Jones & Wang, 2010). Candidate males were assigned to sib groups with 95% confidence and if no suitable candidate males were identified (i.e., not sampled), or unavailable in the case of females, the program reconstructed parental genotypes (Jones & Ardren, 2003). We ran the analysis using the full‐likelihood method with a medium run length and medium likelihood precision as suggest by Jones and Wang (2010) for large datasets. For each year, we ran five replicate runs with different random seeds to assess model convergence and evaluate model uncertainty. This approach has been shown to be more powerful than other methods due to its more efficient use of marker information (Wang & Santure, 2009).

As smallmouth bass have been previously observed to spawn with multiple mates (James, 1930; Neves, 1975; Webster, 1954; Wiegmann & Baylis, 1995; Wiegmann et al., 1992), we therefore assumed a “polygamous” mating system, allowing both full‐ and half‐sib (and unrelated) relationships among fry to be considered. Allele frequencies were updated to account for kin structure within the data, and inbreeding was included in the estimation procedure due to the multigenerational nature of adult sampling (i.e., multiple age classes). To decrease computation times and increase sibship accuracy, we assigned a sibship complexity prior that assumes a multinomial distribution and also provided an average paternal and maternal sibship size prior (i.e., $\overset{¯}{x}$ = 11 offspring/brood). We specified the locus‐specific genotyping (and scoring) error rate, which we obtained by comparing the genotypes of externally tagged males (i.e., T‐bar anchor tags) captured in more than 1 year (values ranged from 0 to 0.0075 depending on locus). The 20 microsatellite loci used in this study were shown to have considerable power to differentiate between the genotypes of sampled individuals based on estimates of the probability of identity described by Waits, Luikart, and Taberlet (2001) and implemented in GIMLET v 1.3.2 (Valière, 2002). The P_(ID)unbiased_ estimates ranging from 1.6E^−9^ (2012 & 2013) to 2.2E^−9^ (2014) and P_(ID)sib_ estimates ranging from 8.2E^−5^ (2013) to 1.1E^−4^ (2014).

### 2.5 Adult spawning participation rate

We used an open‐population, time‐dependent Cormack–Jolly–Seber model (Cormack, 1964; Jolly, 1965; Seber, 1965) implemented in the program MARK (Cooch & White, 2014; White & Burnham, 1999) to determine the annual return rate of nesting adult smallmouth bass and thus infer potential sex‐specific differences in the cost of reproduction. The data consisted of marked and unmarked individuals (based on their multilocus genotype) captured during successive spawning seasons [three seasons (2012–2014) for females; four seasons (2011–2014) for males] and were represented as individual capture histories. Estimates of survival probabilities φ_*ij*_ (i.e., interpreted here as return rate) and their sampling variances were calculated using a maximum likelihood estimation procedure based on a conditional product‐multinomial distribution of recaptures. We did not interpret the return rate estimates strictly as survival because of the difference in duration between this study and the lifespan of the bass in this population. The standard error (SE) and 95% confidence intervals (CI) for each estimate were determined using a profile likelihood approach which takes into account the shape of the distribution of likelihood values. We assumed a perfect probability of detection (*p*_*ij*_ = 1.0) to allow parameter estimates at all‐time steps. We felt this was a reasonable first approximation due to the comprehensive nature of repeated surveys along the entire shoreline coupled with the presumed natal philopatry and strong nest site fidelity observed for this species.

### 2.6 Mate choice or mate selection

We estimated pairwise genetic relatedness (R; Queller & Goodnight, 1989) between all male–female spawning pairs using the package RELATED v1.0 (Pew, Muir, Wang, & Frasier, 2015) in R (R Development Core Team, 2016) to investigate female mate choice and determine whether females actively avoided mating with close relatives or pursued additional spawning partners to whom they were less related than the first male with which they spawned (based on nest phenology data). A relatedness score of −1 represents two maximally dissimilar individuals, a score of 1 indicates individuals having identical genotypes, and a score of zero represents the average relatedness of two randomly chosen individuals in the population. Using the same 20 microsatellite loci to assess parentage and genetic similarity can result in a false‐positive relationship between parentage and genetic similarity; however, this bias is often negligible when using seven or more loci (Wetzel & Westneat, 2009).

We performed randomization tests using the method of Fossøy, Johnsen, and Lifjeld (2007) to determine the significance of pairwise genetic relatedness estimates for each male–female pair. This approach was developed to reduce potential statistical biases that can occur with such comparisons (Wetzel & Westneat, 2009). To test significance, we generated randomized distributions based on the relatedness estimates between each respective female and all nesting males sampled within the South Arm of the lake during each respective year. For scenarios where females spawned with more than one male, the randomized distribution of relatedness values was generated for each respective female and all nesting males; except males with which a female had spawned with previously in the season were excluded in each case. We performed randomization procedures using the software RESAMPLING STATS v4.0 (Simon, 1997) and iterated each test 10,000 times. Significance values were determined by the proportion of iterations that exceeded the value of each male–female pair.

### 2.7 Statistical analysis

We used a general linear model (GLM) to compare the relatedness values of multiple mating females with the first male and any subsequent males with which they spawned within a given season. Models were fit to the data using maximum likelihood procedures described by Zuur, Ieno, Walker, Saveliev, and Smith (2009). We assumed a normal distribution of relatedness scores and used an identity link to model this relationship. We used each female–male triad only once to control for pseudo‐replication. For females that spawned with more than one additional male, we randomly selected only one of these males to include in the analysis. The difference in genetic relatedness between each female and their first and second male was used as the response variable and year was the sole explanatory variable. This allowed us to test whether the difference in relatedness was significantly different from zero. Analyses were performed in R (R Development Core Team, 2016) using the “nlme” package (Pinheiro, Bates, DebRoy, & Sarkar, 2015).

## 3 RESULTS

### 3.1 Pedigree reconstruction

Molecular pedigree analysis based on the 20 microsatellite loci reconstructed full‐ and half‐sib families (i.e., broods) of smallmouth bass with 91.3% (*n* = 400) accuracy (i.e., results consistent with known spatial sampling information for both nest guarding males and their respective broods) (Table 1). Of the 5,013 swim‐up fry samples included in the analysis, 4,339 (86.6%) were correctly assigned to their respective nest guarding male. For the correctly assigned broods, 1.3% of swim‐up fry (*n* = 61) were mis‐assigned to an incorrect male–female pair, 1.7% (*n* = 76) were assigned to an unknown (i.e., not sampled) male–female pair, and 2.1% (*n* = 97) were assigned to a male–female pair from a neighboring nest but had become mixed prior to sampling.

**Table 1:** Reconstructed molecular pedigree data for adult smallmouth bass nesting in the South Arm of Lake Opeongo during the 2012–2014 spawning seasons. The total number of successfully assigned broods and respective number of offspring are provided for each year. The number of individuals from each nest identified as being mixed from a neighboring nest, of unknown origin, or mis‐assigned to the incorrect nest is also provided. Excluded broods include those in which the nest tending male was not captured, all individuals were mixed from a neighboring nest, and all individuals were assigned to an unknown pair, or mis‐assigned to an incorrect spawning pair based on spatial sampling data

	2012				2013				2014			
	Broods	%	Offspring	%	Broods	%	Offspring	%	Broods	%	Offspring	%
Assigned	153	90.53	1,634	85.91	175	91.62	1,886	86.08	72	92.31	819	89.02
Mixed	–	–	28	1.47	–	–	58	2.65	–	–	11	1.20
Unknown	–	–	33	1.74	–	–	31	1.41	–	–	12	1.30
Mis‐assigned	–	–	24	1.26	–	–	30	1.37	–	–	7	0.76
Total	153	90.53	1,719	90.38	175	91.62	2,005	91.51	72	92.31	849	92.28
Excluded												
No male	4	2.37	44	2.31	5	2.62	59	2.69	2	2.56	23	2.50
Mixed	5	2.96	59	3.10	4	2.09	48	2.19	1	1.28	12	1.30
Unknown	4	2.37	46	2.42	4	2.09	44	2.01	2	2.56	24	2.61
Mis‐assigned	3	1.78	34	1.79	3	1.57	35	1.60	1	1.28	12	1.30
Total	16	9.47	183	9.62	16	8.38	186	8.49	6	7.69	71	7.72
Grand Total	169		1,902		191		2,191		78		920	

A total of 38 complete broods were excluded from the analysis. Eleven broods were excluded at the outset because we failed to capture the respective nest guarding male. Ten broods consisted entirely of offspring assigned to a male–female pair from a neighboring nest, and thus, the target nests had likely failed prior to sampling. Ten additional broods were assigned to an unknown male–female pair, which likely represent mixed broods from neighboring nests where the adult male was not sampled or could have resulted from errors in genotyping of the respective nest guarding male. The latter explanation is unlikely, however, as each male was genotyped multiple times. Only seven broods were mis‐assigned to an incorrect nest based on spatial sampling information collected in the field at the time when the swim‐up fry were captured.

### 3.2 Genetic mating system

Smallmouth bass spawning in Lake Opeongo displayed a complex mating system (Table 2). For the successful spawning attempts surveyed, 85.3% of broods (*n* = 341) were produced by a single male receiving a clutch of eggs from a single female, but nearly 12.5% of nests (*n* = 50) received eggs from second female and 2.3% of nests (*n* = 9) received eggs from a third female. Notably, a single male in 2014 was identified as having produced two separate clutches from different females at the same nest location (one early spawning and one late spawning female). Females, on the other hand, were more likely (*n* = 391; 91.1%) to participate in single‐pair mating than males; except for the 2014 breeding season in which a disproportion number of females (*n* = 14; 21.5%) deposited eggs in a second nest, and a single female (1.5%) deposited eggs in a third nest. Lastly, there was no evidence of male cuckoldry (broods assigned to a single female and more than one male within a nest male).

**Table 2:** Mating pattern among adult smallmouth bass nesting within the South Arm of Lake Opeongo during the 2012–2014 spawning seasons. Male mating pattern is represented by the number of broods (and offspring) successfully assigned to the respective attending male for monogamous pairs and nests with multiple females (i.e., polygyny). Mating pattern for reconstructed females in also represented by the number of broods (and offspring) assigned to each monogamous female and females that deposited eggs in multiple nests (i.e., polyandry)

	2012				2013				2014			
	Broods	%	Offspring	%	Broods	%	Offspring	%	Broods	%	Offspring	%
Assigned paternity												
Single Female	128	83.66	1,364	83.48	149	85.14	1,603	84.99	64	88.89	720	87.91
Two females	20	13.07	251	13.40	23	13.14	251	13.31	7	9.72	89	10.87
Three females	5	3.27	32	3.12	3	1.71	32	1.70	1	1.39	10	1.22
Total	153		1,634		175		1,886		72		819	
Reconstructed females												
Single Male	165	94.83	1,490	91.19	176	92.63	1,630	86.43	50	76.92	515	62.88
Two males	9	5.17	144	8.81	14	7.37	256	13.57	14	21.54	277	33.82
Three males	0	0.00	0	0.00	0	0.00	0	0.00	1	1.54	27	3.30
Total	174		1,634		190		1,886		65		819	

**Figure 2:**
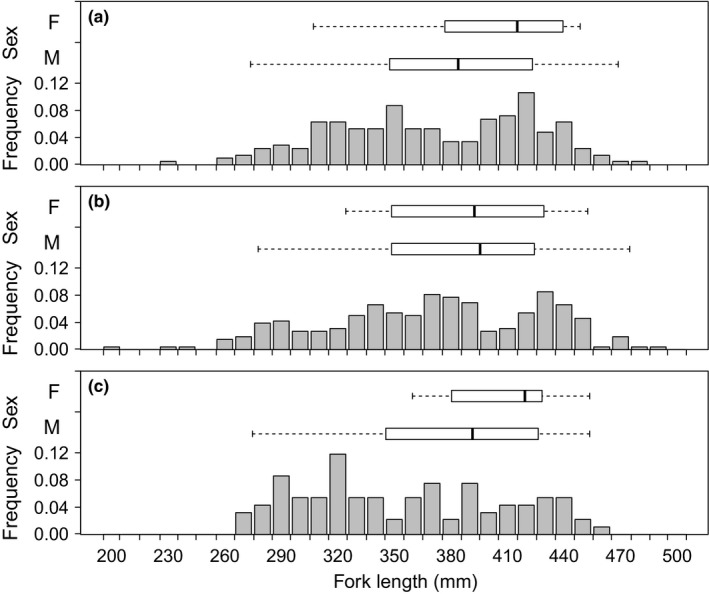
Fork length of first males that spawned with multiple mating females (F) and multiple mating males (M) relative to the size distribution of all males in the South Arm of Lake Opeongo during the (a) 2012, (b) 3013, and (c) 2014 spawning seasons

Multiple mating males were generally larger that single‐pair males but displayed considerable variation among years (Figure 2). The median size of males that received a clutch of eggs from more than one female during the 2012, 2013, and 2014 spawning seasons was 381.0 mm (*n* = 25), 392.5 mm (*n* = 26), and 388.5 mm (*n* = 8), respectively. However, individuals as small as 272 mm (2012) were also observed to receive eggs from more than one female but multiple mate spawning by individuals of this size was relatively rare. Females who deposited eggs in more than one nest were also believed to be generally larger individuals assuming the length of a female's first mate within a season provided a relative indicator of female body size given the occurrence of size assortative mating in this species. The median size of males selected first by multiple mating females was 412.0 mm (*n* = 9), 389.5 mm (*n* = 14), and 416.0 mm (*n* = 15) during the 2012, 2013, and 2014 spawning seasons, respectively. Moreover, first males were never smaller than 322 mm.

### 3.3 Nest adult return rates

The proportion of individuals returning to spawn in more than one season was higher for males than females (Table 3). When examined across all 4 years, a total of 449 unique males were identified to have spawned in the South Arm of Lake Opeongo. Of these males, 304 (68%) were determined to have spawned in a single season whereas 145 (32%) spawned in more than one season. Moreover, 10% of returning males (*n* = 15) were determined to have skipped (i.e., not observed in) one or more years between spawning attempts. When we restricted our data to only those years in which the genotypes of spawning females were reconstructed (2012–2014; *n* = 362 males), a total of 259 nesting males (72%) spawned only once whereas 103 of males (29%) spawned in more than one season. With this restricted dataset, only a single returning male (1%) was determined to have skipped a year between spawning attempts. During this same time period, 398 unique females were determined to have spawned in the surveyed study area. Of these females, 371 (93%) spawned in a single year and 63 (18%) spawned in more than 1 year. Of the returning females, 31 (49%) skipped a year between spawning attempts.

**Table 3:** Number of unique adult males and females spawning in Lake Opeongo during the 2011–2014 spawning seasons beginning with the years each individual was first observed and includes the total number of seasons each individual was observed. The parentheses represent the number of individuals within each category that was observed to skip season(s) between observations

Year of 1st observations	Sex	Number of seasons observed							
		1		2		3		4	
		Number of individuals	Skip	Number of individuals	Skip	Number of individuals	Skip	Number of individuals	Skip
2011	M	45	(0)	19	(4)	17	(10)	6	(0)
	F	–	–	–	–	–	–	–	–
2012	M	95	(0)	66	(1)	15	(0)	–	–
	F	157	(0)	15	(3)	4	(0)	–	–
2013	M	123	(0)	22	(0)	–	–	–	–
	F	164	(0)	8	(0)	–	–	–	–
2014	M	41	(0)	–	–	–	–	–	–
	F	50	(0)	–	–	–	–	–	–

The estimated return rate (φ_ij_) for adult male smallmouth bass nesting in the South Arm of Lake Opeongo was consistently higher than that of spawning females, yet the pattern of change across years was similar among both sexes. The male return rate remained relatively stable between the 2011–2012 (φ_ij_ = 0.38; SE ± 0.06; 95% CI, 0.28 ≤ φ_ij_ ≤ 0.50) and 2012–2013 time intervals (φ_ij_ = 0.46; SE ± 0.04; 95% CI, 0.39 ≤ φ_ij_ ≤ 0.53) but was markedly lower during the 2013–2014 time interval (φ_ij_ = 0.18, SE ± 0.03; 95% CI, 0.14 ≤ φ_ij_ ≤ 0.24). Despite being consistently lower than that of nesting males, the female return rate during the 2012–2013 time interval (φ_ij_ = 0.09; SE ± 0.02; 95% CI, 0.06 ≤ φ_ij_ ≤ 0.15) was only slightly higher than the 2013–2014 time intervals (φ_ij_ = 0.06; SE ± 0.02; 95% CI, 0.04 ≤ φ_ij_ ≤ 0.11).

### 3.4 Multiple mating and mate choice

Mating among nesting adult smallmouth bass in Lake Opeongo was largely random with respect to their multilocus genotype (Table 4). Of the 429 nesting pairs (i.e., female and first males) surveyed during the 2012–2014 spawning seasons, 382 (89%) had relatedness values that did not deviate significantly from random expectations. However, there was evidence of mating between close relatives (i.e., inbreeding) for 25 (6%) male–female pairs, and a similar number of spawning pairs (*n* = 22; 5%) showed evidence of significant outbreeding.

**Table 4:** Relatedness categories of male–female pairs with significance determined based on 10,000 bootstrap iterations of relatedness estimates for each individual female and all potential males spawning in the South Arm of Lake Opeongo during the 2012–2014 breeding seasons including the number and proportion of all male–female pairs within each relatedness category, and the range of relatedness values observed within each category

	2012			2013			2014		
	Pairs	%	Range (*r*_xy_)	Pairs	%	Range (*r*_xy_)	Pairs	%	Range (*r*_xy_)
Inbred	8	0.046	(0.374 to 0.672)	12	0.063	(0.241 to 0.489)	5	0.077	(0.197 to 0.439)
Unrelated	154	0.880	(−0.475 to 0.372)	170	0.895	(−0.538 to 0.419)	58	0.892	(−0.417 to 0.383)
Outbred	13	0.074	(−0.501 to 0.215)	8	0.042	(−0.612 to −0.294)	2	0.015	(−0.439 to −0.325)

**Figure 3:**
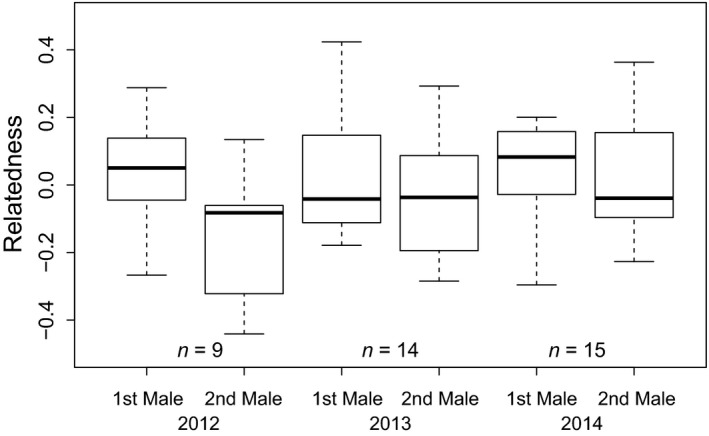
Relatedness of multiple mating females with their first (1st male) and subsequent males (2nd male) for the 2012–2014 spawning seasons in the South Arm of Lake Opeongo. For the female that spawned with more than one additional male in 2014, we randomly selected only one of these males to include in the analysis to control for pseudo‐replication. Sample size (*n*) represents the number of spawning pairs

Within a season, multiple mating females showed some evidence for selecting additional males to which they were genetically less related than the first male with which they spawned (Figure 3), but the signal was generally weak and variable among years. The 2012 spawning season was the only year with significant evidence of additional males being less related to their respective female than first males (ML: *t* = 2.940, *p* = .006, Estimate ± SE: 0.186 ± 0.053, *N* = 9 broods). Despite the direction of the relationship being similar, the difference between multiple mating females and their first and additional males was not significant during the 2013 (ML: *t* = −1.435, *p* = .160, Estimate ± SE: 0.070 ± 0.081, *N* = 14 broods) and 2014 spawning seasons (ML: *t* = −1.940, *p* = .060, Estimate ± SE: 0.031 ± 0.080, *N* = 15 broods).

## 4 DISCUSSION

### 4.1 Genetic mating system

Smallmouth bass are included in a growing list of taxa where social and genetic mating systems differ (Avise et al., 2002). The genetic mating system of smallmouth bass in Lake Opeongo can be characterized as predominantly monogamous with a low rate of polygynandry (see Shuster & Wade, 2003) particularly among larger individuals. Broods produced within a season were largely the result of single‐pair mating (one male and one female) but a small number of individuals from both sexes participated in multiple mating, with males displaying greater variance in mate number than females. Our findings support to some degree the long‐standing belief that members of the *Micropterus* genus represent one of the rare examples of monogamy among freshwater fishes (Avise et al., 2002; DeWoody & Avise, 2001; DeWoody, Fletcher, Wilkins, Nelson, & Avise, 2000; Wiegmann et al., 1992), but the occurrence of multiple mating by both sexes indicates that strict monogamy implied by early analyses of *Micropterus* mating systems is incorrect. Moreover, iteroparous individuals showed a complete absence of mate fidelity from one spawning season to the next. We suspect the absence of interannual mate fidelity in this species may be a result of high mortality among nesting adults presumably due to the high energetic demands (i.e., costs) of reproduction (e.g., Steinhart, Leonard, Stein, & Marschall, 2005). We recognize the current analysis falls short of a full examination of survival and determining whether the observed pattern is the result of a differential cost of reproduction among other factors requires further research.

Multiple mating has long been known to occur in smallmouth bass (James, 1930; Neves, 1975; Webster, 1954; Wiegmann & Baylis, 1995; Wiegmann et al., 1992) but not to the extent observed in Lake Opeongo. Our data revealed that nearly 14.8% of males and 8.9% of females spawned with multiple different mates. This is slightly higher than values reported for largemouth bass (*Micropterus salmoides*), where 12% of nests contained offspring from more than one female (DeWoody et al., 2000) but not to the extent seen in other centrarchid species such as bluegill (Ehlinger, 1997; Gross, 1982) or redbreast sunfish (DeWoody, Fletcher, Wilkins, Nelson, & Avise, 1998). Uniparental care displayed by smallmouth bass provides an opportunity for both males and females to pursue additional mates, and yet the majority of broods produced within any given breeding season were the result of single‐pair mating. This suggests the environmental potential to participate in multiple mating is either nonexistent for these individuals or that behavioral or physiological constraints may limit their ability to take advantage of this potential (Emlen & Oring, 1977; Shuster & Wade, 2003). If true, this could have considerable evolutionary implications as mate number has been suggested to influence total variation in male brood size more than female fecundity (Wiegmann et al., 1992) and therefore may have contributed to the large annual variance in reproductive success previously documented among nesting males in this population (Gross & Kapuscinski, 1997).

The pursuit of multiple mating opportunities by male smallmouth bass appears to be at least partially constrained by the requirements of parental care. The general absence of eggs at different stages of development in the same nest suggests male receptivity may be restricted to a relatively short period of time (Ridgway & Friesen, 1992; Wiegmann et al., 1992). Smallmouth bass exhibit elaborate courtship behavior both away from and at the nest site (Ridgway, 1989), with the entire spawning sequence potentially lasting a few hours (Reighard, 1906; Schneider, 1971). Depredation of broods in untended nests has been shown to occur rapidly in Lake Opeongo, as smallmouth bass in this system experience high predation driven mortality on eggs and larvae (Dunlop, Orendorff, Shuter, Rodd, & Ridgway, 2005; Ridgway & Friesen, 1992). The potential losses to an individual male by withholding care from one set of offspring while courting and mating with additional females could therefore be greater than the gains resulting from such behavior (Emlen & Oring, 1977; Westneat, 1990). Moreover, the intensity of male nest‐defense behavior has been shown to increase with egg and larval development stage (Coble, 1975; Ridgway, 1988), and this change in behavior could deter additional females from spawning with males whose nest already contains eggs from other females. As a result, these males are likely to be unavailable to later spawning females and this could limit a male's ability to take full advantage of the environmental potential for polygamy.

The ability of female smallmouth bass to engage in multiple mating appears to be constrained by the allometric relationship between female body size and fecundity (Raffetto, Baylis, & Serns, 1990; Vogele, 1981; Wiegmann et al., 1992). Research has shown larger females have a higher gonad mass to body mass ratio than smaller females (Ridgway, Shuter, et al. 1991; Ridgway, MacLean, et al. 1991). Therefore, larger females presumably have a greater number of eggs to divide among multiple nests defended by different territorial males. However, female fitness can only be enhanced to the degree that nest guarding males are sexually receptive and willing to assume incubation of these additional clutches (Emlen & Oring, 1977). Asynchrony in the timing of spawning seen among nesting males suggests large, early spawning females are likely to have little difficulty locating additional nests with receptive males, which may not be the case for their smaller, later spawning counterparts (Ridgway & Friesen, 1992; Ridgway, Shuter, et al. 1991). Moreover, males have been shown to adjust their nest‐defense behavior according to the number of offspring for which care is being provided (Ridgway, 1989; Sargent, 1988), and the probability of male abandonment has been shown to be inversely related to brood size (Lunn & Steinhart, 2010; Steinhart & Lunn, 2011). Accordingly, larger females are likely to be more capable of producing a sufficient quantity of eggs to deposit separate clutches in multiple nests without risking premature male abandonment due to small clutch size.

The inability of male smallmouth bass to fully capitalize on the environmental potential for polygamy due to either ecological or behavioral constraints may in part explain the lack of sexual dimorphism or the absence of alternative reproductive tactics (Ridgway, 1989) often seen in other centrarchids (DeWoody et al., 1998; Gross, 1984, 1991; Neff & Clare, 2008; Neff, Fu, & Gross, 2003). Multiple mating is expected to increase the variance in reproductive success among individuals and thus increase the strength of sexual selection (Kempenaers & Schlicht, 2010). However, the predominance of single‐pair mating together with the low annual return rate of spawning adults is likely to limit the lifetime variance in reproductive success of male smallmouth bass and therefore limit the strength of sexual selection. Moreover, multiple mating by females can decrease the sex difference in the opportunity for selection between males and females (Shuster & Wade, 2003). This occurs because multiple mating females partition their eggs among multiple nests and thus into groups of progeny with different paternity. As a consequence, the variance in offspring number among males decreases relative to the case where a single male sires all of the offspring of a particular female and thereby limits the strength of sexual selection (Shuster & Wade, 2003). However, the potential effects of multiple mating on the strength of sexual selection in this and other centrarchid species remain an important area of research.

### 4.2 Female mate choice

The smallmouth bass population in Lake Opeongo does not show elevated levels of inbreeding. This finding runs contrary to the conclusions of Gross et al. (1994) who suggested the similarity of DNA fingerprint banding patterns among progeny within nests and for male–female spawning pairs could be the result of natal philopatry, which is expected to concentrate relatives during reproduction and thus increase the probability of mating between related individuals. If smallmouth bass are truly philopatric, our data suggest kin recognition or other behavioral mechanisms (i.e., movement and dispersal patterns) may be operating to prevent extensive mating between close relatives in this population. Kin recognition has been empirically demonstrated for other centrarchid species (Brown & Colgan, 1986; Hain & Neff, 2006) but has not been previously examined in smallmouth bass. Sex bias in movement and dispersal patterns has long been considered a primary means by which species reduce the probability of mating between close relatives (Dobson, 2013; Greenwood, 1980). Yet despite considerable effort (e.g., Ridgway et al., 2002), natal and breeding dispersal patterns in smallmouth bass are not well understood particularly among females. Alternatively, the low level of genetic diversity observed in this population could reflect levels of genetic variation in the brood source(s) used to establish the population (Christie, 1957; Gross et al., 1994) or be a consequence of the founding process itself (i.e., allee/founder effect, Frankham 1995).

Understanding why females participate in multiple mating is an important topic in evolutionary ecology, and various hypotheses have been proposed (Akçay & Roughgarden, 2007; Kempenaers, 2007; Westneat, 1990). Despite some evidence female smallmouth bass may pursue multiple mating as a possible inbreeding avoidance mechanism (see Arct, Drobniak, & Cichoń, 2015; Brooker, Rowley, Adams, & Baverstock, 1990), this pattern was relatively weak and varied among years. Females may also spawn with multiple males to increase genetic diversity (i.e., heterozygosity) in their offspring (Casey, Sandercock, & Wisely, 2011; Smith, Webster, & Holmes, 2005) or ensure genetic compatibility (e.g., Kempenaers, 2007; Sillero‐Zubiri, Gottelli, & Macdonald, 1996; Stockley, Searle, Macdonald, & Jones, 1993; Tregenza & Wedell, 2000). A positive relationship between fitness and heterozygosity (i.e., heterosis or heterozygote advantage) has been demonstrated in a variety of taxa (Evans & Neff, 2009; Hedrick, 2001) (but see Casey et al., 2011; Kempenaers, 2007). However, empirical studies comparing survivorship among half‐siblings from the same or different broods would be required to assess any potential fitness benefit associated with multiple mating by female smallmouth bass (e.g., Griffith et al., 2002; Kempenaers, Verheyen, & Dhondi, 1997; Neff & Pitcher, 2005; Sheldon, Merilö, Qvarnström, Gustafsson, & Ellegren, 1997). Alternatively, this behavior may represent a bet‐hedging strategy in unstable environments (e.g., Portnoy, Piercy, Musick, Burgess, & Graves, 2007), where females deposit eggs in the nest of other males to prevent against a total loss of reproductive effort due to predation pressure, male abandonment, or storm‐related internal seiche events (MacLean, Shuter, Regier, & MacLeod, 1981; Steinhart & Lunn, 2011).

## 5 SUMMARY

Animal mating systems are extremely diverse, and considerable lability in mating patterns can occur when individuals both within and among populations are exposed to different environmental or demographic conditions. Our findings provide some support for the long‐standing belief that members of the *Micropterus* genus represent one of the rare examples of monogamy among freshwater fishes, but the occurrence of multiple mating by both males and females within the Lake Opeongo smallmouth bass population suggests prior claims of strict monogamy are likely unfounded. Uniparental care provides an opportunity for both sexes to pursue additional mates. And yet, the majority of broods produced within a season were the result of single‐pair mating. This suggests the environmental potential to participate in multiple mating is either nonexistent for most individuals or that behavioral or physiological constraints limit their ability to take advantage of this potential. The inability of male smallmouth bass to fully capitalize on the environmental potential for polygamy due to either ecological or behavioral constraints, together with multiple mating by females, could limit the strength of sexual selection and therefore explain to the lack of sexual dimorphism and the absence of alternative reproductive tactics often seen in other centrarchid species. Our findings provide novel insights into the factors influencing the genetic mating system of smallmouth bass and how they may affect the strength of sexual selection in this species.

## CONFLICT OF INTEREST

None declared.

## AUTHOR CONTRIBUTIONS

Franckowiak R, Ridgway M, and Wilson C were involved in project development, design, analysis and all contributed to the manuscript.

## DATA ACCESSIBILITY

Microsatellite genotype data, biological data, and nest location data available from the Dryad Digital Repository: https://doi.org/10.5061/dryad.cq5h4.

DNA sequences for newly developed microsatellite loci: GenBank accession numbers MF621595–MF621604.
