# Supplementary material for: Genetic mating system and mate selection in smallmouth bass
Source: Ecol Evol. 2017 Sep 20;7(21):8864–75. doi: 10.1002/ece3.3423 (PMC5677493; doi:10.1002/ece3.3423)
Supplement: Supplementary file 1 [file ECE3-7-8864-s001.docx]

**Microsatellite Library Development and Multiplex Optimization**

**Introduction**

Knowledge of genealogical relationships among individuals in natural populations has important research applications in behavioral ecology, evolutionary biology, and conservation (Blouin 2003; Jones & Ardren 2003; Pemberton 2008; Jones & Wang 2010). Such information can be used to assess movement and dispersal patterns (Dow & Ashley 1996; Hardesty et al. 2006), characterize kin-based social structure (Amos et al. 1993), investigate mating patterns (Clapham & Palsboll 1997; Jones & Avise 1997), mate choice (Rudnick et al. 2005), inbreeding (Keller & Weller 2002), and also evaluate the influence of behavioral strategies and life-history tradeoffs on individual variance in reproductive success (Clutton-Brock 1988). Pedigree data also allows for the estimation of quantitative genetic parameters such as trait heritability or genetic correlations between traits (Kruuk et al. 2000; Garant & Kruuk 2005), and provides avenues to investigate how populations are likely to respond to selective pressures in the wild (Merila et al. 2001; Kruuk et al. 2002, 2003; Wilson et al. 2006, 2007).

The utility of genetic markers for determining genealogical relationships has long been realized, but it was not until the discovery of highly polymorphic molecular markers, such as microsatellites (Tautz 1989), that large-scale pedigree analysis was made feasible for natural populations (Blouin 2003; Pemberton 2008); resulting in a flood of empirical studies and the proliferation of statistical methods for assigning parentage (Luikart & England 1999; Jones et al. 2010). Successful implementation of these methodologies requires careful consideration of several issues, but the number and diversity of loci is generally the most important factor determining the accuracy of parentage inference (Bernatchez & Duschesne 2000). A major impediment to conducting parentage analysis in non-model organisms was the development of microsatellite DNA libraries, which was traditionally a labor-intensive and costly process as it required repeat enrichment, cloning, and Sanger sequencing (e.g., Glenn & Schable 2005). Advances in next generation sequencing (NGS) techniques, however, permit the rapid development of microsatellite DNA libraries, and this approach is quickly becoming the preferred method for developing microsatellite markers (Gardner et al. 2011).

Pedigree reconstruction of ecologically and economically important freshwater fish species would greatly expand the range of life history characteristics and breeding systems available for examination (Wilson & Ferguson 2002), and would subsequently provide information essential for their effective management and conservation (Pemberton 2008). Smallmouth bass (*Micropterus dolomieu*) display a number of qualities that make this species particularly relevant for investigating recruitment ecology and reproductive fitness in the wild (Ridgway & Philipp 2002), including male parental care, high fecundity, iteroparity, and plasticity in important life-history traits (Ridgway 1991, Ridgway & Friesen 1992; Dunlop et al. 2005). Previous studies, however, have relied largely on microsatellites developed for closely related species (e.g., Stepien et al. 2007; Borden et al. 2006; Barthel 2010), limiting the number of loci available for conducting parentage analysis. Moreover, for individual-based genetic studies to expand into larger aquatic systems where complete sampling of breeding adults is logistically more difficult, a larger number of markers are needed to ensure accurate parentage assignment (Bernatchez & Duschesne 2000; Wilson & Ferguson 2002; Harrison et al. 2013).

We therefore set out to establish a suite of microsatellite markers suitable for conducting large-scale parentage analysis of smallmouth bass. Using NGS technology to develop a microsatellite DNA library for smallmouth bass, we identified and tested 35 candidate loci from 4,690 contig sequences containing repeat motifs. Ten of these loci consistently amplified and were determined to be polymorphic in this species. We then developed five multiplex reactions containing the 10 newly identified microsatellite loci and 14 additional microsatellite loci which had previously been shown to be polymorphic in smallmouth bass (Malloy et al. 2000; Lutz-Carrillo et al. 2008). Lastly, we examined the characteristics and level of polymorphism of each marker using samples collected from three populations of smallmouth bass in Algonquin Provincial Park, Ontario, Canada.

**Library development**

A single adult smallmouth bass collected from the Otonabee River, Ontario, Canada, was used to generate a genomic DNA library. The sample was prepared as per the standard 454 GS Junior Titanium Sequencing System (454 Life Sciences Corp, Brandford, CT, USA). In short, genomic DNA from muscle and fin tissue was obtained using the DNeasy® blood and tissue protocol (QIAGEN Inc., Valencia, CA, USA), and DNA quantity and quality were checked using a NanoDrop® 1000 spectrophotometer v3.7 (Thermo Fisher Scientific, Waltham, MA, USA). The sample was prepared using a GS FLX Titanium Rapid Library Preparation Kit (Roche Applied Science, Mannheim, Germany) where ~5 ug DNA was fragmented by nebulization prior to the addition of RL adapters and small fragments removed.

The library was then amplified using GS Junior Titanium emPCR Kit (Lib-L) (Roche Applied Science) to produce clonally-amplified, bead-immobilized DNA fragments which were sequenced using the GS Junior Titanium Sequencing kit (Roche Applied Science) and run on a GS Junior Titanium Series instrument (454 Life Sciences Corp). Sequences were trimmed using the default quality clipping points as calculated during signal processing. A total of 139,539 sequence reads were generated which ranged in length from 40 to 676 base pairs (bp) and averaged 453.7 bp. The GS *De Novo* Assembler software (454 Life Sciences Corp) was used to assemble 38,534 sequence reads into 4,690 contig sequences ranging from 100 to 5,785 bp.

Using MSATCOMMANDER v0.8.2 (Faircloth 2008), we searched the assembled contigs to identify sequences containing di-, tri-, and tetranucleotide motif repeats. A total of 186 contigs were identified that contained repeat motifs, but a majority possessed insufficient flanking sequence from which to develop suitable primers. From those that contained sufficient flanking sequence, we were able to design primers for 35 uninterrupted microsatellites using PRIMER3 v1.1.1(Rozen & Skaletsky 2000), with the program criteria set to identify potential primer sets with product sizes ranging from 100 to 350 bp, primer lengths ranging between 18 and 24 bp, annealing temperatures falling between 48°C and 63°C, with primers having between 40% and 60% GC content, and each primer containing at least a single (3’) GC clamp.

**Primer testing and multiplex development**

We used the Henegariu et al. (1997) step-by-step protocol for primer testing and subsequent multiplex reaction development for both the newly identified primers sets and for selected primer sets previously shown to be polymorphic in smallmouth bass (Malloy et al. 2000; Lutz-Carrillo et al. 2008). PCR amplifications (10 μl) were performed on a Mastercycler® Pro thermal cycler (Eppendorf Canada, Mississauga, ON) and fluorescently labelled PCR products were analyzed on an ABI 3730xl DNA Analyzer (Applied Biosystems, Inc., Burlington, ON). After initial testing, we discontinued using loci that were monomorphic or amplified poorly. We proceeded to develop multiplex reactions for a total of 10 newly identified loci along with 14 additional loci selected from the published literature (Table S1). Genetic diversity characteristics were based on a total of 143 smallmouth bass samples collected from Speckled Trout Lake (n = 21), Gordon Lake (n = 38), and Lake Opeongo (n = 84) during the 2011 field season.

Allelic diversity (A_D_) along with observed (H_O_) and expected heterozygosity (H_E_) values were calculated for each marker using GENALEX 6.5 (Peakall & Smouse 2012). To account for differences in sample size, we estimated allelic richness (A_R_) using a rarefaction procedure implemented in HP-RARE (Kalinowski 2005). We tested each population and each locus within each respective population for significant departures from Hardy–Weinberg equilibrium (HWE) and assessed whether there was evidence for linkage disequilibrium (LD) between pairs of loci using GENEPOP v4.5.1 (Rousset 2008). HWE and LD simulations were run with 100,000 dememorization steps, with exact probabilities estimated from 500 batches with 50,000 iterations per batch. Significance was determined following a Sequential Bonferroni adjustment (Holm 1979) to control the family-wise error rate due to multiple comparisons. Exclusion probabilities were calculated using the method of (Jamieson & Taylor 1997) implemented in CERVUS v3.0.7 (Kalinowski et al. 2007).

**Results**

The 24 microsatellite loci generated consistent amplification products with 2–7 alleles per locus and H_O_ values ranging from 0.05 to 0.71 when averaged across all three populations (Table S1). Smallmouth bass from Speckled Trout Lake had noticeably lower levels of genetic diversity in terms of number of alleles per locus, although, this site also had the smallest sample size (Table S2). After correcting for sample size, Lake Opeongo had an average allelic richness value (A_R_ = 2.55), intermediate between Speckled Trout Lake (A_R_ = 1.99) and Gordon Lake (A_R_ = 3.13) based on a minimum sample size of 40 genes/alleles. A similar pattern was observed for mean observed heterozygosity with Speckled Trout Lake (H_O_ = 0.30) exhibiting lower heterozygosity levels than Lake Opeongo (H_O_ = 0.38), which again exhibited lower levels of heterozygosity than Gordon Lake (H_O_ = 0.47).

All three populations conformed to HWE expectations (data not shown). One locus (Mdo10) showed a significant departure from HWE expectations in all three populations due to significant heterozygote deficiency, and was eliminated from the data set. Three loci (MiSaTPW012, MiSaTPW112 and MdoR016) were shown to depart from HWE expectations in Lake Opeongo at the nominal alpha level (α = 0.05), but were not significant following sequential Bonferroni correction. All other loci in all three populations conformed to HWE expectations. All loci also conformed to linkage equilibrium expectations with the exception of MiSaTPW173 and MiSaTPW181, which exhibited significant linkage when averaged across all three populations and in two (Gordon Lake and Lake Opeongo) of the three populations when tested separately. Locus MiSaTPW181 was therefore eliminated from the dataset to avoid interpretation bias. Two additional loci (Mdo9 and MiSaTPW157) were removed due to a lack of polymorphism observed in samples collected from Lake Opeongo.

The remaining 20 loci were deemed suitable for large-scale pedigree analysis of the Lake Opeongo smallmouth bass population. Based on levels of polymorphism for these loci, the combined exclusion probabilities for an unrelated candidate parent when one candidate parent is unknown, an unrelated candidate parent when both candidate parents are known, and a pair of unrelated candidate parents of an arbitrary offspring were calculated to be 0.9899, 0.9998, and 0.9999, respectively.

**Literature Cited**

Amos B, Schlotterer C, Tautz D (1993) Social structure of pilot whales revealed by analytical DNA profiling. *Science*, 260, 670-672.

Barthel BL (2010) Influence of dispersal, natal environment, and variance in reproductive success on the genetic relationships within a population of freshwater fish. PhD dissertation. School of Integrative Biology. University of Illinois at Urbana-Champaign.

Bernatchez L, Duchesne P (2000) Individual-based genotype analysis in studies of parentage and population assignment: how many loci, how many alleles? *Canadian Journal of Fisheries and Aquatic Sciences*, 57, 1-12.

Blouin MS (2003) [DNA-based methods for pedigree reconstruction and kinship analysis in natural populations](https://scholar.google.ca/citations?view_op=view_citation&hl=en&user=SjapLywAAAAJ&citation_for_view=SjapLywAAAAJ:u5HHmVD_uO8C). *Trends in Ecology and Evolution*, 18, 503-511.

Borden WC, Stepien CA (2006) Discordant population genetic structuring of smallmouth bass, *Micropterus dolomieu* Lacepède, in Lake Erie based on mitochondrial DNA sequences and nuclear DNA microsatellites. *Journal of Great Lakes Research*, 32, 242-257.

Clapham PJ, Palsboll PJ (1997) Molecular analysis of paternity shows promiscuous mating in female humpback whales (*Megaptera novaeangliae*, Borowski). *Proceeding of the Royal Society of London B: Biological Sciences*, 264, 95-98.

Clutton-Brock TH (1988). Reproductive Success. In *Studies of Individual Variation in Contrasting Breeding Systems* (ed. Clutton-Brock TH), pp. 538. The University of Chicago Press, Chicago.

Dow BD, Ashley MV (1996) Microsatellite analysis of seed dispersal and parentage of saplings in bur oak, *Quercus macrocarpa*. *Molecular Ecology*, 5, 615-627.

Dunlop ES, Orendorff JA, Shuter BJ, Rodd FH, Ridgway MS (2005) Diet and divergence of introduced smallmouth bass (*Micropterus dolomieu*) populations. *Canadian Journal of Fisheries and Aquatic Sciences*, 62, 1720-1732.

Faircloth BC (2008) MSATCOMMANDER: detection of microsatellite repeat arrays and automated, locus-specific primer design. *Molecular Ecology Resources*, 8, 92-94.

Garant D, Kruuk LEB (2005) How to use molecular marker data to measure evolutionary parameters in wild populations. *Molecular Ecology*, 14, 1843-1859.

Gardner MG, Fitch AJ, Bertozzi T, Lowe AJ (2011) Rise of the machines – recommendations for ecologists when using next generation sequencing for microsatellite development. *Molecular Ecology Resources*, 11, 1093-1101.

Glenn TC, Schable, NA (2005) Isolating microsatellite DNA loci. *Methods in Enzymology*, 395, 202-222.

Hardesty BD, Hubbell SP, Bermingham E (2006) Genetic evidence of frequent long-distance recruitment in a vertebrate-dispersed tree. [*Ecology Letters*](https://www.researchgate.net/journal/1461-0248_Ecology_Letters), 9, 516-525.

Harrison HB, Saenz-Agudelo P, Planes S, Jones GP, Berumen ML (2013) Relative accuracy of three common methods of parentage analysis in natural populations. *Molecular Ecology*, 22, 1158-1170.

Henegariu O, Heerema NA, Dlouhy SR, Vance GH, Vogt PH (1997) Multiplex PCR: Critical parameters and step-by-step protocol. *BioTechniques*, 23, 504-511.

Holm S (1979) A simple sequentially rejective multiple test procedure.*Scandinavian Journal of Statistics*, 6, 65-70.

Jamieson A, Taylor StCS (1997) Comparisons of three probability formulae for parentage exclusion. *Animal Genetics*, 28, 397-400.

Jones AG, Avise JC (1997) Microsatellite analysis of maternity and the mating system in the Gulf pipefish, *Syngnathus scovelli*, a species with male pregnancy and sex-role reversal. *Molecular Ecology*, 6, 203-213.

Jones AG, Ardren WR (2003) [Methods of parentage analysis in natural populations](https://scholar.google.ca/citations?view_op=view_citation&hl=en&user=Bg0HqUQAAAAJ&citation_for_view=Bg0HqUQAAAAJ:u5HHmVD_uO8C). *Molecular ecology*, 12, 2511-2523.

Jones OR, Wang J (2010) Molecular marker-based pedigrees for animal conservation biologists. *Animal Conservation*, 13, 26-34

Jones AG, Small CM, Paczolt KA, Ratterman NL (2010) A practical guide to methods of parentage analysis. *Molecular Ecology Resources*, 10, 6-30.

Kalinowski ST (2005) HP-Rare: A computer program for performing rarefaction on measures of allelic diversity. *Molecular Ecology Notes*, 5, 187-189.

Kalinowski ST, Taper ML, Marshall TC (2007) Revising how the computer program CERVUS accommodates genotyping error increases success in paternity assignment. *Molecular Ecology*, 16, 1099-1106.

Keller LF, Waller DM (2002) Inbreeding effects in wild populations. *Trends in Ecology and Evolution*, 17, 230-241.

Kruuk LEB, Clutton-Brock TH, Slate J, Pemberton JM, Brotherstone S, Guinness FE (2000) Heritability of fitness in a wild mammal population. Proceedings of the National Academy of Science USA, 97, 698-703.

Kruuk LEB, Merila J. Sheldon BC (2003) When environmental variation short circuits natural selection. *Trends in Ecology and Evolution*, 18, 207-209.

Kruuk LEB, Slate J, Pemberton JM, Brotherstone S, Guinness F, Clutton-Brock TH (2002) Antler size in red deer: heritability and selection but no evolution. *Evolution*, 56, 1683-1695.

Luikart G, England PR (1999) Statistical analysis of microsatellite DNA data. *Trends in Ecology and Evolution*, 14, 253-256.

Lutz-Carrillo DJ, Hagen C, Dueck LA, Glenn TC (2008) Isolation and characterization of microsatellite loci for Florida largemouth bass, *Micropterus salmoides floridanus*, and other micropterids. *Molecular Ecology Resources*, 8, 178-184

Malloy TP, Van Den Bussche RA, Coughlin WD, Echelle AA (2000) Isolation and characterization of microsatellite loci in smallmouth bass, *Micropterus dolomieu* (Teleostei : Centrarchidae), and cross-species amplification in spotted bass, *M. punctulatus*. *Molecular Ecology*, 9, 1946-1948.

Merila J, Kruuk LEB, Sheldon BC (2001) Cryptic evolution in a wild bird population. *Nature*, 412, 76-79.

Peakall R, Smouse PE (2012) GenAlEx 6.5: genetic analysis in Excel. Population genetic software for teaching and research – an update. *Bioinformatics*, 28, 2537-2539.

Pemberton JM (2008) Wild pedigrees: the way forward. *Proceeding of the Royal Society of London B: Biological Sciences*, 275, 613-621.

Ridgway MS, MacLean JA, MacLeod JC (1991) Nest site fidelity in a centrarchid fish, the smallmouth bass (*Micropterus dolomieu*). *Canadian Journal of Zoology*, 69, 3103-3105.

Ridgway MS, Friesen TG (1992) Annual variation in parental care in smallmouth bass, *Micropterus dolomieu*. *Environmental Biology of Fishes*, 35, 243-255.

Ridgway MS, Philipp D (2002). Current status and future directions for research in the ecology, conservation and management of black bass in North America. In: *Black Bass: Ecology, Conservation and Management*, Symposium 31 (ed. Philipp DP, Ridgway MS), pp. 719-724. American Fisheries Society, Bethesda, Maryland.

Rousset F (2008) genepop’007: a complete re-implementation of the genepop software for Windows and Linux. *Molecular Ecology Resources*, 8, 103-106.

Rozen S, Skaletsky, H (2000) Primer3 on the WWW for general users and for biologist programmers. In: *Bioinformatics Methods and Protocols: Methods in Molecular Biology* (ed. Krawetz S, Misener S), pp. 365-386. Humana Press, Totowa, New Jersey.

Rudnick JA, Katzner TE, Bragin EA, Rhodes OE, DeWoody JA (2005) Using naturally shed feathers for individual identification, genetic parentage analyses, and population monitoring in an endangered eastern imperial eagle (*Aquila heliaca*) population from Kazakhstan. *Molecular Ecology*, 14, 2959-2967.

Stepien CA, Murphy DJ, Strange RM (2007) Broad- to fine-scale population genetic patterning in the smallmouth bass *Micropterus dolomieu* across the Laurentian Great Lakes and beyond: an interplay of behaviour and geography. *Molecular Ecology*, 16, 1605-1624.

Tautz D (1989) Hypervariability of simple sequences as a general source for polymorphic DNA markers. *Nucleic Acids Research*, 17, 6463-6471.

Wilson AJ, Ferguson MM (2002) Molecular pedigree analysis in natural populations of fishes: approaches, applications, and practical considerations. *Canadian Journal of Fisheries and Aquatic Sciences*, 59, 1696-1707.

Wilson A, Pemberton J, Pilkington J, Coltman D, Mifsud D, Clutton-Brock TH, Kruuk L (2006) Environmental coupling of selection and heritability limits phenotypic evolution. *PLoS Biology*, 4, 1270-1275.

Wilson AJ, Pemberton JM, Pilkington JG, Clutton-Brock TH, Coltman DW, Kruuk LEB (2007) Quantitative genetics of growth and cryptic evolution of body weight in an island population. *Evolutionary Ecology*, 21, 337-356.

| Multiplex | Locus | Label | Primer  (uM) | KCl buffer | BSA (mM) | dNTPs (mM) | MgCl_2_ (mM) | T_m_ (°C) | N | Repeat | Range (bp) | A_D_ | H_O_ | GenBank  Accession |
| --- | --- | --- | --- | --- | --- | --- | --- | --- | --- | --- | --- | --- | --- | --- |
| 1 | Mdo9 | FAM | 0.08 | 1.0x | 1.0 | 0.4 | 1.5 | 58 | 143 | (GT)_10_ | 126-130 | 2 | 0.08 | AF294497 |
|  | MiSaTPW130 | PET | 0.17 |  |  |  |  |  | 141 | (AG)_12_ | 231-259 | 6 | 0.34 | EF590100 |
|  | MiSaTPW157 | NED | 0.08 |  |  |  |  |  | 143 | (AC)_21_ | 135-153 | 4 | 0.17 | EF590106 |
|  | MiSaTPW165 | FAM | 0.14 |  |  |  |  |  | 142 | (AC)_16_ | 242-260 | 4 | 0.43 | EF590108 |
|  | MiSaTPW173 | VIC | 0.09 |  |  |  |  |  | 143 | (AC)_15_ | 177-195 | 6 | 0.68 | EF590112 |
| 2 | Mdo3 | NED | 0.11 | 1.0x | 1.0 | 0.4 | 1.8 | 58 | 143 | (CA)_20_ | 118-124 | 3 | 0.48 | AF294491 |
|  | MiSaTPW012 | NED | 0.11 |  |  |  |  |  | 143 | (AGAT)_21_ | 276-292 | 6 | 0.47 | EF590067 |
|  | MiSaTPW107 | VIC | 0.08 |  |  |  |  |  | 143 | (AC)_12_ | 246-274 | 7 | 0.47 | EF590091 |
|  | MiSaTPW112 | FAM | 0.09 |  |  |  |  |  | 142 | (AG)_13_ | 175-197 | 7 | 0.70 | EF590094 |
|  | MiSaTPW116 | PET | 0.08 |  |  |  |  |  | 143 | (AC)_21_ | 196-206 | 4 | 0.46 | EF590096 |
| 3 | Mdo1 | FAM | 0.07 | 1.0x | 1.0 | 0.4 | 1.4 | 53 | 143 | (GT)_14_ | 195-211 | 4 | 0.36 | AF294489 |
|  | Mdo10 | VIC | 0.08 |  |  |  |  |  | 140 | (GT)_10_ | 99-105 | 3 | 0.02 | AF294498 |
|  | MiSaTPW025 | VIC | 0.14 |  |  |  |  |  | 143 | (AGAT)_11_ | 283-339 | 4 | 0.54 | EF590071 |
|  | MiSaTPW181 | PET | 0.13 |  |  |  |  |  | 143 | (AC)_22_ | 339-359 | 3 | 0.28 | EF590113 |
| 4 | MdoR007 | VIC | 0.18 | 1.0x | 1.0 | 0.3 | 1.8 | 58 | 143 | (CA)_12_ | 106-130 | 6 | 0.58 | MF621595 |
|  | MdoR016 | FAM | 0.15 |  |  |  |  |  | 141 | (AC)_14_ | 166-198 | 4 | 0.67 | MF621596 |
|  | MdoR033 | VIC | 0.2 |  |  |  |  |  | 143 | (AC)_18_ | 202-228 | 4 | 0.34 | MF621597 |
|  | MdoR037 | PET | 0.16 |  |  |  |  |  | 143 | (GT)_14_ | 115-135 | 6 | 0.38 | MF621598 |
|  | MdoR050 | NED | 0.13 |  |  |  |  |  | 142 | (AC)_13_ | 187-205 | 4 | 0.60 | MF621599 |
| 5 | MdoR005 | VIC | 0.15 | 1.0x | 1.0 | 0.3 | 1.5 | 51 | 143 | (GT)_13_ | 147-173 | 5 | 0.71 | MF621600 |
|  | MdoR009 | PET | 0.18 |  |  |  |  |  | 143 | (CA)_11_ | 153-155 | 2 | 0.26 | MF621601 |
|  | MdoR015 | FAM | 0.16 |  |  |  |  |  | 143 | (CA)_13_ | 207-225 | 4 | 0.27 | MF621602 |
|  | MdoR022 | FAM | 0.09 |  |  |  |  |  | 143 | (TC)_13_ | 134-138 | 2 | 0.05 | MF621603 |
|  | MdoR028 | PET | 0.11 |  |  |  |  |  | 143 | (CA)_12_ | 179-181 | 2 | 0.26 | MF621604 |

Table S1. Details for five microsatellite multiplex reactions developed for smallmouth bass including locus identifier, florescent label, primer concentration, KCl buffer concentration, BSA concentration, dNTP concentration, MgCl_2_ concentration, annealing temperature (T_m_), sample size (N), repeat motif, allele size range in base pairs, allelic diversity (A_D_), observed heterozygosity (H_O_), and GenBank accession number. Locus characteristics averaged for Speckled Trout Lake, Gordon Lake and Lake Opeongo.

|  | Speckled Trout Lake | | | | | |  | Gordon Lake | | | | | |  | Lake Opeongo | | | | | |
| --- | --- | --- | --- | --- | --- | --- | --- | --- | --- | --- | --- | --- | --- | --- | --- | --- | --- | --- | --- | --- |
| Locus | N | A_D_ | A_R_ | H_O_ | H_E_ | HWE |  | N | A_D_ | A_R_ | H_O_ | H_E_ | HWE |  | N | A_D_ | A_R_ | H_O_ | H_E_ | HWE |
| Mdo9 | 42 | 1 | 1.00 | 0.00 | 0.00 | --- |  | 76 | 2 | 2.00 | 0.29 | 0.29 | 1.00 |  | 168 | 1 | 1.00 | 0.00 | 0.00 | --- |
| MiSaTPW130 | 40 | 2 | 2.00 | 0.30 | 0.33 | 1.00 |  | 76 | 5 | 4.88 | 0.55 | 0.63 | 0.59 |  | 166 | 2 | 2.00 | 0.25 | 0.24 | 1.00 |
| MiSaTPW157 | 42 | 1 | 1.00 | 0.00 | 0.00 | --- |  | 76 | 3 | 2.53 | 0.47 | 0.37 | 0.19 |  | 168 | 2 | 1.81 | 0.07 | 0.07 | 1.00 |
| MiSaTPW165 | 40 | 2 | 2.00 | 0.55 | 0.45 | 0.61 |  | 76 | 3 | 3.00 | 0.61 | 0.61 | 0.66 |  | 168 | 3 | 2.56 | 0.32 | 0.32 | 0.70 |
| MiSaTPW173 | 42 | 4 | 4.00 | 0.71 | 0.75 | 0.33 |  | 76 | 3 | 3.00 | 0.74 | 0.67 | 0.71 |  | 168 | 4 | 3.95 | 0.64 | 0.67 | 0.20 |
| Mdo3 | 42 | 2 | 2.00 | 0.48 | 0.46 | 1.00 |  | 76 | 2 | 2.00 | 0.55 | 0.51 | 0.75 |  | 168 | 3 | 2.92 | 0.45 | 0.55 | 0.13 |
| MiSaTPW012 | 42 | 3 | 2.95 | 0.48 | 0.50 | 1.00 |  | 76 | 4 | 3.85 | 0.50 | 0.48 | 0.86 |  | 168 | 3 | 3.00 | 0.45 | 0.48 | 0.02 |
| MiSaTPW107 | 42 | 3 | 3.00 | 0.62 | 0.64 | 0.81 |  | 76 | 3 | 2.76 | 0.13 | 0.17 | 0.26 |  | 168 | 4 | 3.99 | 0.58 | 0.65 | 0.58 |
| MiSaTPW112 | 42 | 2 | 2.00 | 0.52 | 0.44 | 0.61 |  | 74 | 4 | 3.91 | 0.59 | 0.63 | 0.74 |  | 168 | 5 | 4.99 | 0.80 | 0.77 | 0.04 |
| MiSaTPW116 | 42 | 2 | 2.00 | 0.52 | 0.47 | 0.66 |  | 76 | 4 | 3.98 | 0.63 | 0.67 | 0.16 |  | 168 | 2 | 2.00 | 0.37 | 0.43 | 0.30 |
| Mdo1 | 42 | 1 | 1.00 | 0.00 | 0.00 | --- |  | 76 | 3 | 3.00 | 0.74 | 0.64 | 0.57 |  | 168 | 2 | 2.00 | 0.27 | 0.24 | 0.35 |
| Mdo10 | 42 | 2 | 2.00 | 0.00 | 0.09 | 0.02 |  | 76 | 3 | 2.78 | 0.08 | 0.22 | 0.00 |  | 162 | 1 | 1.00 | 0.00 | 0.00 | --- |
| MiSaTPW025 | 42 | 2 | 1.95 | 0.05 | 0.05 | --- |  | 76 | 4 | 3.98 | 0.79 | 0.71 | 0.94 |  | 168 | 3 | 2.42 | 0.55 | 0.50 | 0.75 |
| MiSaTPW181 | 42 | 2 | 1.95 | 0.05 | 0.05 | --- |  | 76 | 3 | 3.00 | 0.63 | 0.49 | 0.07 |  | 168 | 2 | 1.99 | 0.18 | 0.16 | 1.00 |
| MdoR007 | 42 | 2 | 2.00 | 0.43 | 0.44 | 1.00 |  | 76 | 5 | 4.53 | 0.84 | 0.76 | 0.86 |  | 168 | 5 | 4.09 | 0.50 | 0.48 | 0.74 |
| MdoR016 | 42 | 2 | 2.00 | 0.48 | 0.50 | 1.00 |  | 76 | 4 | 3.98 | 0.84 | 0.68 | 0.12 |  | 164 | 3 | 2.96 | 0.65 | 0.56 | 0.04 |
| MdoR033 | 42 | 2 | 2.00 | 0.57 | 0.50 | 0.66 |  | 76 | 4 | 3.89 | 0.50 | 0.48 | 0.96 |  | 168 | 2 | 2.00 | 0.21 | 0.21 | 1.00 |
| MdoR037 | 42 | 3 | 2.95 | 0.24 | 0.35 | 0.20 |  | 76 | 4 | 3.68 | 0.55 | 0.57 | 0.72 |  | 168 | 3 | 2.67 | 0.35 | 0.43 | 0.11 |
| MdoR050 | 42 | 2 | 2.00 | 0.52 | 0.49 | 1.00 |  | 76 | 4 | 4.00 | 0.82 | 0.69 | 0.57 |  | 166 | 3 | 2.97 | 0.52 | 0.54 | 0.76 |
| MdoR005 | 42 | 3 | 3.00 | 0.48 | 0.61 | 0.25 |  | 76 | 4 | 3.95 | 0.79 | 0.69 | 0.36 |  | 168 | 3 | 3.00 | 0.74 | 0.65 | 0.34 |
| MdoR009 | 42 | 2 | 2.00 | 0.10 | 0.09 | 1.00 |  | 76 | 1 | 1.00 | 0.00 | 0.00 | --- |  | 168 | 2 | 2.00 | 0.42 | 0.46 | 0.47 |
| MdoR015 | 42 | 1 | 1.00 | 0.00 | 0.00 | --- |  | 76 | 3 | 3.00 | 0.39 | 0.36 | 1.00 |  | 168 | 2 | 2.00 | 0.29 | 0.28 | 1.00 |
| MdoR022 | 42 | 1 | 1.00 | 0.00 | 0.00 | --- |  | 76 | 1 | 1.00 | 0.00 | 0.00 | --- |  | 168 | 2 | 1.86 | 0.08 | 0.08 | 1.00 |
| MdoR028 | 42 | 1 | 1.00 | 0.00 | 0.00 | --- |  | 76 | 2 | 1.53 | 0.03 | 0.03 | --- |  | 168 | 2 | 2.00 | 0.43 | 0.36 | 0.14 |

Table S2. Levels of genetic diversity in three smallmouth bass populations for 24 microsatellite loci including sample size (N), allelic diversity (A_D_), allelic richness (A_R_), observed (H_O_) and expected heterozygosity (H_E_), and Hardy-Weinberg Equilibrium exact probability tests (HWE).
